# Individuals living with lupus: findings from the LUPUS UK Members Survey 2014

**DOI:** 10.1177/0961203317749746

**Published:** 2018-01-08

**Authors:** C Morgan, A R Bland, C Maker, J Dunnage, I N Bruce

**Affiliations:** 1 Arthritis Research UK Centre for Epidemiology, Centre for Musculoskeletal Research, School of Biological Sciences, Faculty of Biology, Medicine and Health, The University of Manchester, Manchester, UK; 2 National Institute for Health Research (NIHR) Manchester Musculoskeletal Biomedical Research Centre, Central Manchester University Hospitals NHS Foundation Trust, Manchester Academic Health Science Centre, Manchester, UK; 3 428245 LUPUS UK, St James House, Romford, UK

**Keywords:** LUPUS UK, patient experience, SLE, survey

## Abstract

Systemic lupus erythematosus (SLE) is a complex and unpredictable disease which varies greatly among patients and has a significant impact on an individual’s daily living and quality of life. A better understanding of the patients’ experiences with the disease is vital to the effective management of the disease. LUPUS UK, a national UK-registered charity supporting people with systemic and discoid lupus, conducted a UK-wide survey of individuals living with lupus in order to provide foundation information to support and identify gaps needing further research.

An anonymous survey was sent to 5660 LUPUS UK members in order to obtain demographic, diagnosis, symptom and treatment information. A total of 2527 surveys were returned by 2371 females (mean age 56.9 years, SD 13.6) and 156 males, (mean age 60.9 years, SD 15.7). Individuals reported a mean (SD) time to diagnosis from the first symptom of 6.4 (9.5) years, with 47% (*n* = 1186) initially being given a different diagnosis prior to lupus. Fatigue/weakness (91%, *n* = 2299) and joint pain/swelling (77.4%, *n* = 1957) were the most common symptoms that interfere with daily activities, while 73% (*n* = 1836) noted having some problems that make them unable to carry out their usual daily activities. Thirty-two per cent (*n* = 806) were also seeking support beyond traditional pharmacological treatments, such as acupuncture and massage. This study highlights the range and frequency of symptoms difficult to live with on a daily basis and support areas needing further research to improve patients’ well-being.

## Introduction

Systemic lupus erythematosus (SLE) is a multisystem inflammatory disease caused by dysregulation of the immune system with contribution from genetic, hormonal and environmental factors. The disease mainly affects young females, with a 9:1 ratio of those diagnosed being female.^1^ SLE is more prevalent in people of African ancestry as well as Indo-Asians and individuals from the Far East such as China.^2^

Diagnosing SLE can be difficult due to the complex and unpredictable disease course, as well as the evolution of clinical features developing over time. In general, there is also no single measure that can confirm the diagnosis.^3^ Whilst some individuals may be mildly affected, others can experience severe relapses of life-threatening disease. Early recognition of SLE can lead to better treatment and preventive care, thereby avoiding the more severe and long-standing outcomes such as renal impairment, cardiovascular disease, and infections.^4^ Quality of life and the individual personal burden of SLE are equally important to allow us to better understand the illness from the patient’s perspective. Symptoms of SLE include pain, fatigue, sleep disturbances, depression and psychological disorders with considerable emotional adjustment often required.^5^ Whilst health care providers may focus more on symptoms and signs that reflect specific organ involvement and/or damage and other co-morbidities, patients often highlight more the symptoms that affect their everyday lives.^6^ The measurement of outcomes and experiences with the disease from the patient’s perspective is clinically meaningful^6^ and therefore it is essential to consider when planning services and developing a research agenda.

Further insight is needed into the personal burden of SLE in everyday living and to identify areas of unmet need from the patient’s perspective. LUPUS UK, a national United Kingdom (UK)-registered charity supporting people with systemic and discoid lupus, performed a nationwide survey of lupus and individuals with manifestations of lupus to provide foundation information to identify gaps needing further research and to give a better overall understanding of the personal impact of living with SLE in the UK.

## Methods

### Survey population and data collection

LUPUS UK developed a survey questionnaire in collaboration with The Arthritis Research UK Centre for Epidemiology, University of Manchester (see Appendix 1 in supplementary material). Survey questions included: month and year of birth, gender, ethnicity (based on Office for National Statistics 2011 Census for England and Wales), living status and where applicable, details of work status and benefit support. Individuals were asked details of their consultant diagnosis(es) including month and year and details of other diagnoses they may have previously been given for this condition. Participants were asked to indicate the symptoms that regularly affect them, those they found more difficult to live with and conditions making their symptoms worse, such as sunlight, cold/damp, fatigue and stress. Information on general health including problems with mobility, self-care, anxiety/depression, pain and fatigue and level of disruption to undertake usual activities were also indicated. The level of support individuals received and any beneficial treatment including both prescribed and alternative therapies were also noted. Ethical approval from the local ethics committee was confirmed not to be required given the anonymous methods of data collection and transfer to the university.

### Statistical methods

The demographic and survey responses were summarized using descriptive statistics expressed as mean ± standard deviation (SD) or as percentages where appropriate using STATA 13.

## Results

### Demographic characteristics

The survey was mailed to 5660 registered members of LUPUS UK, of whom 44.6% (*n* = 2527) were completed and returned. Of respondents, 93.8% (*n* = 2371) were female and 6.2% (*n* = 156) were male, with a mean (SD) age at survey date of 56.9 (13.6) years and 60.9 (15.7) years, respectively. The majority of the respondents were of White British ethnicity (93%, *n* = 2350) with 2.7% (*n* = 68) and 1.8% (*n* = 46) of African and Indo-Asian origin, respectively. Regarding home circumstances, 78.9% (*n* = 1994) lived with their partner or family and 19.4% (*n* = 489) lived alone (supplementary Table 1). Only 15% (*n* = 378) of individuals worked full time with 51.1% (*n* = 1291) receiving some form of benefits and the majority having retired (52.8%, *n* = 1335). Of retirees, 44.0% (*n* = 587) had retired on medical grounds (supplementary Table 2).

### Diagnosis characteristics

Patients could indicate what main diagnosis(es) they had been given. The most frequent diagnosis was SLE (88%) with 68.3% having SLE as their sole diagnostic term. Other reported additional diagnoses or aspects of their disease included discoid lupus (10.6%), ‘connective tissue disease’ (CTD) (11.7%), lupus nephritis (9.5%) and antiphospholipid syndrome (APS)/Hughes’ syndrome (9.7%) (supplementary Table 3).

The mean (SD) reported time from the first ‘lupus’ symptom to diagnosis was 6.4 (9.5) years (Table 1). This mean (SD) time was shorter in men (4.8 (8.7) years) (Table 1) and Black patients (2.8 (3.6) years) (supplementary Table 4). Examining the time to diagnosis by decade of when the diagnosis was made, no trend was observed, indicating no temporal trend towards earlier time to diagnosis in recent decades (Table 1).

**Table 1:** Self-reported time to diagnosis from first symptom by decade of diagnosis

Time to diagnosis (years) mean (SD)	Pre-1990 (*n* = 461)		1990–1999 (*n* = 737)		2000–2009 (*n* = 772)		2010–2014 (*n* = 350)		Total (*n* = 2320)	
5.8 (7.9)		6.5 (9.9)		6.5 (10.1)		6.4 (8.8)		6.4 (9.5)	
**Males**	**Females**	**Males**	**Females**	**Males**	**Females**	**Males**	**Females**	**Males**	**Females**
	5.5 (9.1)	5.8 (7.8)	5.2 (10.7)	6.6 (9.8)	3.9 (6.5)	6.7 (10.4)	4.4 (6.7)	6.5 (8.9)	4.8 (8.7)	6.5 (9.5)

One-half of respondents had been given a different diagnosis prior to their final diagnosis (46.9%, *n* = 1186). The most common were rheumatoid arthritis (RA; 36.7%, *n* = 435), chronic fatigue (16.1%, *n* = 191), ‘skin disorders’ (11.2%, *n* = 133) and a chronic psychological disorder (10.8%, *n* = 128). In total, chronic fatigue, fibromyalgia and psychological problems contributed to almost one-third (26.9%, *n* = 319) of all prior diagnoses (supplementary Tables 5 and 6).

The commonest co-morbidities reported were thyroid disease (12.9%, *n* = 326), overlapping RA (11.2%, *n* = 283), osteoporosis (9.0%, *n* = 227), osteoarthritis (10.1%, *n* = 256) and cardiac conditions (11.5%, *n* = 291) (supplementary Table 7).

### Symptoms experienced

**Figure 1:**
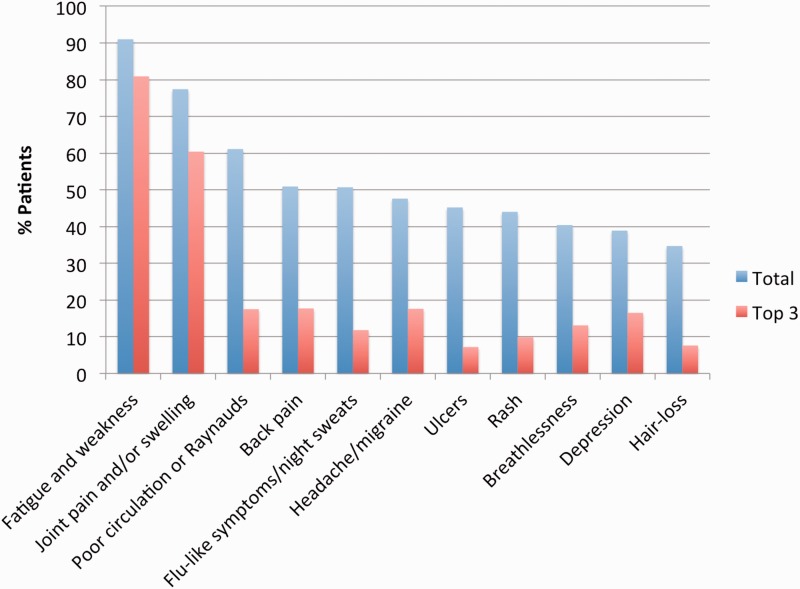
Most frequently reported symptoms to suffer from stratified by total number of participants experiencing a symptom and number of participants ranking symptoms in the top three of most difficult symptoms to live with.

A total of 53.8% (*n* = 1360) patients reported suffering frequently from between 6 and 10 different symptoms. Fatigue and weakness were experienced by 91.0% (*n* = 2299) of the group and 77.4% (*n* = 1957) had regular joint pain or swelling (Figure 1). When asked to rank the top three symptoms most difficult to live with, these first two symptoms were also ranked in their ‘top three’ by 80.9% (*n* = 2044) and 60.4% (*n* = 1527) of individuals, respectively (supplementary Tables 8 and 9).

Conditions reported to make lupus worse both in males and females were over-exertion and fatigue (males; 96.2% *n* = 108, females; 82.6%, *n* = 1959), followed by stress and worry (males; 52.6% *n* = 82, females; 75.2% *n* = 1784,), sunlight (males; 43% *n* = 67, females; 57.6% *n* = 1365), and cold and damp conditions (males; 44.8% *n* = 70, females; 56.2% *n* = 1333) (supplementary Table 10).

General health indicators on the day of the survey also suggested poor health of the group, with 19.7% (*n* = 498) having problems walking or being in a wheelchair, 27.3% (*n* = 689) reporting extreme pain and discomfort, 19.9% (*n* = 502) experiencing extreme anxiety or depression and 72.7% (*n* = 1836) having some problems or unable to carry out their usual activities (Table 2).

**Table 2:** General health indicators on the day of survey completion

General health variable *n* (%)	Men *n* = 156	Women *n* = 2371	Total *n* = 2527
Mobility
No problems	59 (37.8)	892 (37.6)	951 (37.6)
Some problems	57 (36.5)	974 (41.1)	1031 (40.8)
Problem walking/in wheelchair	37 (36.5)	461 (19.4)	498 (19.7)
Housebound	1 (0.6)	32 (1.4)	33 (1.3)
Self-care
No problems	111 (71.2)	1540 (65.0)	1651 (65.3)
Some problems	39 (25.0)	715 (30.2)	754 (29.8)
Unable to/extreme	5 (3.2)	93 (3.9)	98 (3.9)
Carry out usual activities
No problems	56 (35.9)	665 (28.1)	721 (28.5)
Some problems	72 (46.2)	1271 (53.6)	1343 (53.2)
Unable to/extreme	26 (16.7)	423 (17.8)	449 (17.8)
Problems with pain/discomfort
No problems	31 (19.9)	240 (10.1)	271 (10.7)
Some problems	91 (58.3)	1464 (61.8)	1555 (61.5)
Unable to/extreme	32 (20.5)	657 (27.7)	689 (27.3)
Anxiety/depression
No problems	54 (34.6)	575 (24.3)	629 (24.9)
Some problems	64 (41.0)	1270 (53.6)	1334 (52.8)
Unable to/extreme	29 (18.6)	473 (20.0)	502 (19.9)
Pain from lupus in past week (VAS 0–10) mean (SD)	3.7 (3.0)	4.8 (2.8)	4.7 (2.9)
Fatigue from lupus in past week (VAS 0–10) mean (SD)	5.1 (3.3)	6.4 (2.7)	6.3 (2.8)

VAS: visual analogue scale.

### Health care and support

In 82.5% (*n* = 2085) of individuals, their rheumatologist was reported as their current lead health care professional for clinical management, with 16.9% (*n* = 426) and 11.3% (*n* = 285) seeing a dermatologist and nephrologist, respectively (supplementary Table 11). Using a visual analogue scale, patients reported high levels of day-to-day support from their partners, family and friends in addition to their treating consultant (Table 3).

**Table 3:** Support an individual received (VAS scale 0–10) by gender group

Support mean (SD)	Men *n* = 156	Women *n* = 2371	Total *n* = 2527
Partner	9.0 (2.4)	7.9 (3.1)	7.2 (3.2)
Consultant	8.1 (2.8)	7.6 (2.7)	7.7 (2.7)
Family	7.6 (3.6)	7.2 (3.2)	7.2 (3.2)
Friends	5.9 (3.7)	6.3 (3.2)	6.3 (3.3)
GP	6.8 (3.2)	6.2 (3.4)	6.2 (3.4)
Other specialist	6.1 (3.8)	5.5 (3.8)	5.5 (3.8)
Specialist nurse	5.9 (3.8)	5.2 (4.0)	5.3 (4.0)
LUPUS group^a^	4.7 (3.8)	5.2 (3.8)	5.2 (3.8)

a Excluding those indicating did not use.

VAS: visual analogue scale; GP: general practitioner.

### Treatment

Patients self-reported high levels of therapy use with the commonest being glucocorticoids (67%, *n* = 1694) and anti-malarials (67.9%, *n* = 1715) with 45.1% (*n* = 1140) also taking immunosuppressants (Table 4).

**Table 4:** Frequency of self-reported medication use by gender

Medication *n* (%)	Men *n* = 156	Women *n* = 2371	Total *n* = 2527
Steroids	96 (61.5)	1598 (67.4)	1694 (67.0)
Anti-malarials	91 (58.3)	1624 (68.5)	1715 (67.9)
Immunosuppressant	77 (49.4)	1063 (44.8)	1140 (45.1)
Biologics	11 (7.1)	117 (4.9)	128 (5.1)
Non-steroidals	48 (30.8)	859 (36.2)	907 (35.9)
Other medication	31 (19.9)	433 (18.3)	464 (18.4)
Alternative therapy	14 (9.0)	460 (19.4)	474 (18.8)

**Figure 2:**
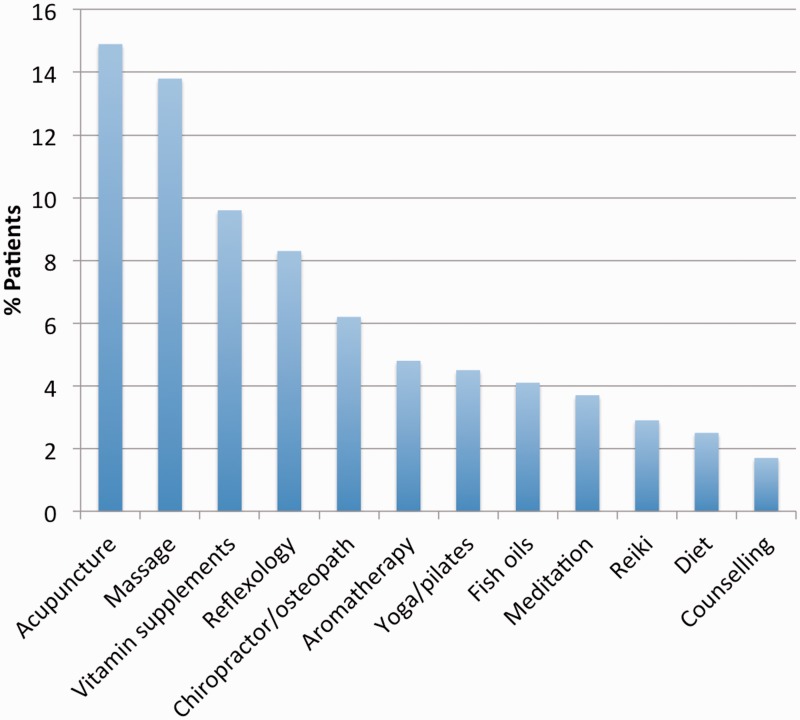
Alternative therapies sought by patients.

Regarding alternative or complementary therapies, 32% (*n* = 806) accessed these, with acupuncture and massage being the most common (14.9%, *n* = 120 and 13.8%, *n* = 111, respectively) (Figure 2).

## Discussion

This UK-wide survey of individuals living with SLE provides important insights into the personal burden of the disease in everyday living and highlights areas for further research. First, we found there was a significant period of time (6.4 years) between initial symptoms and reaching the diagnosis of SLE. Almost one-half (46.9%) of the individuals who responded had also been given a different initial diagnosis prior to their final diagnosis. In addition, we found no change in this time to diagnosis over recent decades despite wider awareness of the condition and efforts to improve the identification and diagnosis of SLE. Of interest, the time to diagnosis was much shorter in Black patients (2.8 years). This may reflect the higher incidence and better awareness amongst health care professionals of the possibility of SLE in Black individuals and also since the initial presentation in this population may be more severe, a more prompt diagnosis is also more likely. We also noted a trend towards a shorter time to diagnosis in men (4.8 years). Men in the UK are known to have lower primary care consultation rates than women^7^ and we hypothesize that men may also present with more definite and/or severe symptoms thus prompting earlier referral and diagnosis.

The diagnosis and early recognition of a multisystem disease such as this is a challenge. Patients’ symptomatology evolves over time and in many patients it may be reasonable to conclude they have a different condition early in their course such as RA or a non-specific skin disorder. In addition, many very early features may be non-specific and individually will not raise the possibility of the diagnosis. A recent UK study showed that in the five years prior to diagnosis there is a higher consultation rate amongst patients who are eventually diagnosed as SLE. However, not all consultation could be attributed to features of SLE and many were non-specific such as fatigue and arthralgias.^8^ Our survey noting a time to diagnosis of more than six years found that this time remains steady in recent decades suggesting further work is needed to raise awareness of SLE and related conditions both amongst health care professionals in the UK and amongst patients to recognize the early symptoms and signs of the disease.

Once the diagnosis is confirmed, there is a need to address symptoms that are important to the individual patient including fatigue/weakness and joint pain. In the context of a high reported use of standard therapies, these observations demonstrate that many of the symptoms are not fully addressed by usual treatments. From a patient perspective, commonly used treatments in SLE are falling short of managing the disease and some may even be contributing to residual symptoms.^9^ The shortfall in conventional therapy controlling symptoms is also reflected in 32% of patients reporting use of alternative therapies including acupuncture, massage and reflexology. Further research is therefore needed to focus on potential causes of these symptoms and also to develop newer, more effective, therapies. In addition, more consultation time per patient may be needed to monitor and address potential exacerbating factors for these symptoms. Establishing new biological and non-biological therapies remains the subject of intense ongoing research. Assessing additional adjunctive therapies is also likely to have a role to play in the lupus population as a whole. For instance, there is evidence that low-dose dietary supplementation with omega-3 fish oils in SLE may have a therapeutic effect on disease activity and may also improve endothelial function and reduce oxidative stress.^10^ Likewise, the addition of vitamin D supplementation to standard SLE therapy may improve disease activity and ameliorate inflammatory and haemostatic markers and may also have favourable effects on cardiovascular risk.^11,12^ Others have noted improvements with exercise regimens in SLE, a modality not specifically enquired about in this survey.^13^ Complementary and alternative medicines (CAM) are not considered to be part of conventional medicine yet there is a need to develop an evidence base to support those with true efficacy in SLE disease management.^14^

We found almost three-quarters of individuals had problems that limit their ability to carry out their usual daily activities. Indeed, only 15% of individuals worked full time with over 50% receiving some form of benefits and almost one-half of retirees retiring on medical grounds. This highlights that SLE symptoms impact directly on an individual’s ability to maintain employment, contributing to a high societal burden across both direct health costs and indirect costs of social care. At an individual level, many patients also require day-to-day support not only from health care professionals but also from a partner, family member and friends. A holistic approach is therefore required which encompasses regular support from health care professionals, psychological support such as counselling, exercise, diet and evidence-based alternative therapies.

This survey highlights the range and frequency of symptoms difficult to live with on a daily basis and support areas needing further research to improve patients’ well-being. SLE is a complex disease with multiple presentations and with numerous symptoms that may overlap with other conditions. Clinical management involves consideration of the interaction of lupus drugs and medications for other conditions. Thus careful monitoring both of clinical and patient-reported outcomes is key to better supporting individuals with SLE and managing the impact on their daily lives effectively. Health care providers should aim to treat the aspects of disease that have a more direct effect on individual’s quality of life and furthermore consider the impact upon quality of life when making treatment decisions.

We recognize limitations of this survey. For instance, self-reported patient recall is not formally valid for diagnosis, although the complicated nature of the disease and the nature of the support group suggest it is likely that patients completing the questionnaire do have SLE or some variant of the disease. In particular, the distribution of therapies used is similar to other UK cohorts.^15^ In addition, individuals who participated were more likely to be motivated individuals than individuals declining. As such there is likely to be some bias in those who responded which we could not address, as there was no access to the characteristics of the non-respondents. Nevertheless, the distribution of symptoms and limitations described were similar to observations from more formal studies.^16–18^ Individuals were members of a patient support group, already a motivated cohort potentially better informed about their disease. The ethnic mix of the sample was not fully representative of the UK SLE population. In a recent survey of the incidence and prevalence of SLE in the UK (1999–2012), individuals of Black Caribbean ethnicity had a significantly higher incidence of SLE (31.46/100,000) in comparison to White individuals (6.73/100,000).^19^ Nevertheless the differences found by our survey between different ethnic groups as well as between gender groups are still likely to be valid overall. We accept however that more research and better communication is therefore needed in, for example, Afro-Caribbean, Chinese and Indo-Asian populations in which this disease is more common.

In summary, our survey of a large contemporary UK SLE population shows that the diagnosis of SLE remains a challenge with a long time from early symptoms before making a formal diagnosis and many patients having alternative diagnoses made prior to SLE being confirmed. We also note a significant unmet need in controlling symptoms important to patients such as pain and fatigue. Residual symptoms contribute to significant limitations in daily living, work loss and a need for ongoing support from others as well as seeking unproven complementary therapies. This survey suggests a clear agenda for further patient-focused research to improve awareness of SLE, identify better, more effective therapies and improve support for patients to improve overall quality of life.

## Supplementary Material

Supplementary material

Supplementary material
