# Supplementary material for: Individuals living with lupus: findings from the LUPUS UK Members Survey 2014
Source: Lupus. 2018 Jan 8;27(4):681–7. doi: 10.1177/0961203317749746 (PMC5888773; doi:10.1177/0961203317749746)
Supplement: Supplementary material [file LupusUKQuestionnaireV72014.pdf]

LUPUS UK Survey

One of the main aims of LUPUS UK is to raise awareness of this condition. This survey is to collect information from patients about the effects of lupus so we are asking you to help.

All information is anonymous and no individual can be identified from the information provided. This survey may also be completed online at www.lupusuk.org.uk/survey

Should you have any questions, please contact Chris Maker or Paul Howard on 01708 731251

Thank you very much for your help and support

What is today's date? [ ] day /month/year

About you

1 Gender [ ] male [ ] female

2 Month and year of birth [ ] month year

3 Ethnic group

Please tick which group best describes you

- [ ] White
- [ ] Black-African
- [ ] Black-Caribbean
- [ ] Black-British
- [ ] Black-other
- [ ] Indian
- [ ] Pakistani
- [ ] Bangladeshi
- [ ] Chinese
- [ ] Other Please specify [ ]

4 Country of birth [ ]

5 Who do you live with? [ ] partner/family [ ] friends [ ] alone

About your diagnosis

1 What diagnosis has your consultant given?

Please tick appropriate box(es)

- [ ] SLE
- [ ] Discoid lupus
- [ ] Lupus Nephritis
- [ ] Mixed connective tissue disease
- [ ] Other, please specify: [ ]
- [ ] Awaiting diagnosis

2 In which year did you experience your first symptoms? ☐

3 When were you diagnosed? ☐☐☐☐☐☐

Month Year

4 What age were you when you were diagnosed? ☐

5 Before you were diagnosed with lupus, did any specialist diagnose your symptoms as another condition?

☐ yes ☐ no

If yes, what was the diagnosis?

If necessary, please list other diagnoses here:

6 Do you have any other health conditions?

Please list other conditions you may have:

About your symptoms

1 What symptoms or features regularly affect you?

Please tick any that apply

☐	Rash	☐	Fatigue & weakness	☐	Haematological problems incl anaemia
☐	Mouth ulcers	☐	Flu-like symptoms/ night sweats	☐	Poor circulation or Raynauds
☐	Hair loss	☐	Shortness of breath	☐	Miscarriage (recent or past)
☐	Pain & swelling in joints	☐	Back pain	☐	Kidney problems
☐	Headaches/migraine	☐	Depression	☐	Stroke or mini stroke
				☐	Angina

2 Which three of the above symptoms do you find most difficult to live with?

Please rank in order, the most difficult first:

**1.**
**2.**
**3.**

3 What do you find makes your lupus symptoms worse?

Please tick any that apply:

☐	Sunlight	☐	Over exertion/fatigue
☐	Fluorescent lighting/compact light bulbs	☐	Stress/worry
☐	Heat	☐	Hormonal changes
☐	Cold/damp	☐	Noise

☐ Other, please specify: ______

Your general health

For each of the five activities below, please tick one statement which best describes your own health state. 1 to 3 relate to TODAY.

1 Mobility

Please tick **one** box

- I have no problems in walking ☐
- I have some problems in walking ☐
- I have a problem in walking and need to use a stick/wheelchair ☐
- I am house-bound ☐

2 Self care

Please tick **one** box

- I have no problems with self care ☐
- I have some problems washing and dressing ☐
- I am unable to wash or dress without assistance ☐

3 Usual activities (eg work, study, housework, family/leisure activities)

Please tick **one** box

- I have no problems performing activities ☐
- I have some problems performing my usual activities ☐
- I am unable to perform many normal activities ☐

4 Pain/discomfort

Please tick **one** box

- I have no pain or discomfort ☐
- I have moderate pain or discomfort some days ☐
- I have extreme pain or discomfort most of the time ☐

5 Anxiety/depression

Please tick **one** box

- I am not anxious or depressed ☐
- I am sometimes anxious or depressed ☐
- I am frequently anxious or depressed ☐

6 How much pain have you had because of lupus in the PAST WEEK?

Please mark on the line to indicate the severity of the pain

No pain 0 |—|—|—|—|—|—|—|—|—|—| 10 Severe pain

7 How much of a problem has fatigue or tiredness been in the PAST WEEK?

Please mark on the line to indicate the severity of the fatigue and tiredness

Fatigue is no problem 0 |—|—|—|—|—|—|—|—|—|—| 10 Fatigue is a major problem

Support

1 Which specialist do you currently see or have previously seen?

Please tick any that apply

Current specialist(s)	Specialist(s) previously seen
☐ Rheumatologist	☐ Rheumatologist
☐ Dermatologist	☐ Dermatologist
☐ Nephrologist	☐ Nephrologist
☐ Paediatrician	☐ Paediatrician
☐ Immunologist	☐ Immunologist
☐ Neurologist	☐ Neurologist

2 Please indicate on each scale how active the support you have received has been from each person or group.

	No support (0)	Full support (10)
GP	0	10
Consultant	0	10
Other medical specialist	0	10
Nurse	0	10
Spouse/partner	0	10
Children/other family members	0	10
Friends	0	10
LUPUS UK/local group	0	10

Treatments

1 What treatments have been beneficial to you?

Please tick any that apply

- ☐ Non-steroidals (e.g. Aspirin)
- ☐ Anti-malarials (e.g. Hydroxychloroquine)
- ☐ Steroids (e.g. Prednisolone)
- ☐ Immunosuppressants (e.g. Azathioprine, Methotrexate)
- ☐ Biologics (e.g. Rituximab)
- ☐ Complementary therapy(ies), please specify: ____
- ☐ Other, please specify: ____

Your work status

1 What best describes your current status?

Please tick any box that best describes you

- ☐ full time paid work
- ☐ part-time paid work
- ☐ full time in the home
- ☐ studying full time
- ☐ studying part-time
- ☐ retired due to ill health
- ☐ retired due to age
- ☐ receiving benefits *(If yes, please complete the following question)*

2 If you receive benefits, which of the following do you receive?

Please tick any that apply, indicating which type of allowance and at what level of support you receive where relevant

☐ Disability Living Allowance (DLA)

- ☐ Mobility ☐ High ☐ Low
- ☐ Care ☐ High ☐ Medium ☐ Low

☐ Personal Independence Payments (PIP)

- ☐ Mobility ☐ High ☐ Low
- ☐ Care ☐ High ☐ Low

☐ Incapacity Benefit

☐ Employment & Support Allowance (ESA) ☐ Support Group ☐ Work-Related Activity Group

☐ Attendance Allowance ☐ High ☐ Low

☐ State pension

Other benefits: ____________________

Thank you for completing this survey.

Please return the completed survey in the Freepost envelope provided.

You do not need to use a stamp.
