# Supplementary material for: Individuals living with lupus: findings from the LUPUS UK Members Survey 2014
Source: Lupus. 2018 Jan 8;27(4):681–7. doi: 10.1177/0961203317749746 (PMC5888773; doi:10.1177/0961203317749746)
Supplement: Supplementary material [file SuppMaterialRevision_Oct_24th_2017_clean.pdf]

Supplementary Material

Supplementary Table 1: Demographic data from 2527 individuals included in analysis

**Demographic variable**		**Men n=156 (6.2%)**	**Women n=2371 (93.8%)**	**Total n=2527**
Age at survey date (yrs) mean (SD)		60.9 (15.7)	56.9 (13.6)	57.1 (13.8)
Age group at survey n(%)	0-15	1 (0.6)	2 (0.08)	3 (0.1)
	16-25	3 (1.9)	44 (1.9)	47 (1.9)
	26-35	8 (5.1)	117 (4.9)	125 (5.0)
	36-45	15 (9.6)	292 (12.3)	307 (12.2)
	46-55	16 (10.3)	554 (23.4)	570 (22.6)
	56-65	39 (25.0)	623 (26.3)	662 (26.2)
	66-75	44 (28.2)	555 (23.4)	599 (23.7)
	75+	30 (19.2)	184 (7.7)	214 (8.5)
Age at 1st diagnosis (yrs) mean (SD)		44.0 (16.4)	40.6 (14.0)	40.8 (14.1)
Age group at 1st diagnosis n(%)	0-15	6 (3.9)	50 (2.1)	56 (2.2)
	16-25	19 (12.2)	323 (13.6)	342 (13.5)
	26-35	24 (15.4)	496 (20.9)	520 (20.6)
	36-45	25 (16.0)	611(25.8)	636 (25.2)
	46-55	40 (25.6)	503 (21.2)	543 (21.5)
	56-65	31 (19.9)	269 (11.4)	300 (11.9)
	66-75	5 (3.2)	74 (3.1)	79 (3.1)
	75+	5 (3.2)	15 (0.6)	20 (0.8)
	Did not indicate	1 (0.6)	30 (1.3)	21 (1.2)
Ethnicity n(%)	White	137 (87.8)	2213 (93.3)	2350 (93.0)
	Black-African	0	9 (0.4)	9 (0.4)
	Black-Caribbean	3	32 (1.4)	35 (1.2)
	Black-British	0	21 (0.9)	21 (0.8)
	Black-other	0	3 (0.1)	3 (0.1)
	Indian	7 (4.5)	33 (1.4)	40 (1.6)
	Pakistani	0	5 (0.2)	5 (0.2)
	Bangladeshi	0	1 (0.04)	1 (0.04)
	Chinese	0	14 (0.6)	14 (0.6)
	White Asian	2 (1.3)	8 (0.3)	10 (0.4)
	White Caribbean	0	7 (0.3)	7 (0.3)
	Other mixed race	2 (1.3)	8 (0.3)	10 (0.4)
	Other	0	2 (0.08)	2 (0.08)
	Did not indicate	5 (3.2)	15 (0.6)	20 (0.8)
Living status n(%)	Partner/family	132 (84.6)	1862 (78.5)	1994 (78.9)
	Friend	1 (0.6)	31 (1.3)	32 (1.3)
	Alone	22 (14.1)	467 (19.7)	489 (19.4)
	Did not indicate	1 (0.6)	11 (0.5)	12 (0.5)

Supplementary Table 2: Work status and benefits by gender

Work & benefit status n (%)		Men n=156	Women n=2371	Total n=2527
Working full time paid		32/156 (20.5)	346/2371 (14.6)	378/2527 (15.0)
Working part-time paid		14/156 (9.0)	468/2371 (19.7)	482/2527 (19.1)
Working in the home		9/156 (5.8)	281/2371 (11.9)	290/2527 (11.5)
Studying	Full-time	3/156 (1.9)	21/2371 (0.9)	24/2527 (1.0)
	Part-time	0	30/2371 (1.3)	30/2527 (1.2)
Retired	Medical grounds	39/156 (25.0)	548/2371 (23.0)	587/2527 (23.2)
	Age	61/156 (39.1)	687/2371 (29.0)	748/2527 (29.6)
Receiving benefits		76 (48.7)	1215/2527 (51.2)	1291/2527 (51.1)
Of those receiving benefits:				
Claiming DLA		33/76 (43.4)	688/1215 (55.9)	721/1291 (55.9)
- DLA care (n=605)		28/33 (84.9)	577/688 (83.9)	605/721 (83.9)
- level of care benefit:	Low	8/28 (28.6)	249/577 (43.2)	257/605 (42.5)
	Intermediate	11/28 (39.3)	179/577 (31.0)	210 (26.0)
	High	8/28 (28.6)	135/577 (23.4)	159 (19.7)
	Did not indicate	1/28 (3.6)	14/577 (18.3)	150 (18.6)
- DLA mobility (n=610)		31/33 (93.9)	579/688 (84.2)	610 /721 (84.6)
- level of mobility benefit:	Low	4/31 (12.9)	72/579 (12.4)	76/610 (12.5)
	High	25/31 (80.7)	491/579 (84.8)	516/610 (84.6)
	Did not indicate	2/31 (6.5)	16/579 (2.8)	18/610 (3.0)
Claiming Personal Independence Payments		1/76 (1.3)	42/1215 (3.5)	43/1291 (3.3)
- Mobility (n=28)		1/1 (100)	27/42 (64.3)	28/43 (65.1)
- Level of mobility benefit	Low	0	5/27 (18.5)	5/28 (17.9)
	High	1/1 (100)	20/27 (74.1)	21/28 (75.0)
	Did not indicate	0	2/27 (7.4)	2/28 (7.1)
- Care (n=32)		1/1 (100)	31/42 (73.8)	32/43 (74.4)
- Level of care benefit	Low	0	17/31 (54.8)	17/32(53.1)
	High	0	7/31 (22.6)	7/32 (21.9)
	Did not indicate	1/1 (100)	7/31 (22.6)	8/32 (25.0)
Claiming Incapacity benefit		3/76 (4.0)	82/1215 (6.8)	85/1291 (6.6)
Employment & Support Allowance (n=270)		12/76 (15.8)	258/1215 (21.2)	270 (20.6)
- Support group		7/12 (58.3)	152/258 (58.9)	159/270 (58.9)
- Work-related activity group		2/12 (16.7)	46/258 (17.8)	48/270 (17.8)
- Did not indicate		3/12 (25.0)	60/258 (23.3)	63/270 (23.3)
Claiming Attendance allowance		10/76 (13.2)	78/1215 (6.4)	88/1291 (6.8)
Of those claiming (n=88) Level:	Low	3/10 (30.0)	33/78 (42.3)	36/88 (40.9)
	High	7/10 (70.0)	39/78 (50.0)	46/88 (52.3)
	Did not indicate	0	6/78 (7.7)	6/88 (6.8)
State pension		49/76 (64.5)	602/1215 (49.6)	651/1291 (50.3)

Supplementary Table 3: Diagnosis by gender of 2527 individuals included in analysis

**Diagnosis**	**Men n=156**	**Women n=2371**	**Total n=2527**
Diagnosis n (%)*			
SLE	122 (78.2)	2093 (88.3)	2215 (87.7)
Discoid lupus	27 (17.3)	241 (10.2)	268 (10.6)
CTD	10 (6.4)	286 (12.1)	296 (11.7)
Nephritis	25 (16.0)	216 (9.1)	241 (9.5)
Other disease terms:	16 (10.3)	310 (13.1)	326 (12.9)
Lupus	0	18 (0.8)	18 (0.7)
Cerebral lupus	0	5 (0.2)	5 (0.2)
Lupus-like disease	1 (0.6)	6 (0.3)	7 (0.3)
UCTD	1 (0.6)	4 (0.2)	5 (0.2)
Overlaps	1 (0.6)	14 (0.6)	15 (0.6)
Other cutan.lupus	1 (0.6)	14 (0.6)	14 (0.6)
Anticoag/sticky blood	1 (0.6)	16 (0.7)	17 (0.6)
APS/Hughes	12 (7.7)	232 (9.8)	244 (9.7)
Other CTD diagnosis	1 (0.6)	11 (0.5)	12 (0.5)
Waiting diagnosis	1 (0.6)	13 (0.6)	14 (0.5)
Diagnosis combinations n (%):			
SLE only	97 (62.2)	1628 (68.7)	1725 (68.3)
Discoid only	15 (9.6)	83 (3.5)	98 (3.9)
CTD only	2 (1.3)	71 (3.0)	73 (2.9)
Nephritis only	7 (4.5)	16 (0.7)	23 (0.9)
SLE & discoid	2 (1.3)	97 (4.1)	99 (3.9)
SLE & CTD	2 (1.2)	135 (5.7)	137 (5.4)
SLE & nephritis	11 (7.1)	153 (6.5)	164 (6.5)
Discoid & CTD	3 (1.9)	8 (0.3)	11 (0.4)
Discoid & nephritis	0	1 (0.04)	1 (0.04)
CTD & nephritis	0	2 (0.08)	2 (0.08)
SLE, discoid & CTD	1 (0.6)	19 (0.8)	20 (0.8)
CTD, nephritis & discoid	0	1 (0.04)	1 (0.04)
Nephritis, SLE & discoid	3 (1.9)	12 (0.5)	15 (0.6)
SLE, nephritis, CTD	1 (0.6)	16 (0.7)	17 (0.7)
SLE, nephritis, discoid, CTD	1 (0.6)	6 (0.3)	7 (0.3)

* Patients may have multiple diagnoses’

Supplementary Table 4: Time to diagnosis by ethnicity

	**White n=2161**	**Black-Caribbean n=31**	**Black-British n=21**	**Black-African n=8**	**Black-other n=2**	**Black – All N = 62**	**Indian n=38**	**Chinese n=13**	**White-Asian n=8**	**Pakistani n=4**	**Other mixed race n=9**
Time to diagnosis (yrs) (From 1st symptom diagnosis)	6.5(9.6)	5.5 (6.9)	2.2 (3.5)	1.5 (2.7)	2.03 (1.4)	2.81 (3.63)	3.0 (4.5)	4.4 (6.4)	5.8 (7.1)	0.8 (1.5)	10.2 (12.0)

Supplementary Table 5: Most frequently reported initial diagnoses

**Diagnosis** n (%)	**Men** n=156	**Women** n=2371	**Total** n=2527
Number given different diagnosis prior to final diagnosis	**69 (44.2)**	**1117 (47.1)**	**1186 (46.9)**
Rheumatoid Arthritis	26 (37.7)	409 (36.6)	435 (36.7)
CFS/ME/Fibromyalgia	8 (11.6)	183 (16.4)	191 (16.1)
Skin problems	12 (17.4)	121 (10.8)	133 (11.2)
Psychological problems	3 (4.3)	125 (11.2)	128 (10.8)
Blood related problems	3 (4.3)	57 (5.1)	60 (5.1)
Viral	7 (10.1)	91 (8.1)	98 (8.3)

CFS: Chronic fatigue syndrome; ME: Myalgic encephalomyelitis

Supplementary Table 6: Previous diagnosis by gender

Diagnosis n (%)	Men<br>n=156	Women<br>n=2371	Total<br>n=2527
Number given different diagnosis prior to final diagnosis	**69 (44.2)**	**1117 (47.1)**	**1186 (46.9)**
Rheumatoid	26 (37.7)	409 (36.6)	435 (36.7)
CFS/ME/Fibromyalgia	8 (11.6)	183 (16.4)	191 (16.1)
Skin problems	12 (17.4)	121 (10.8)	133 (11.2)
Psychological problems	3 (4.3)	125 (11.2)	128 (10.8)
Blood related problems	3 (4.3)	57 (5.1)	60 (5.1)
Viral	7 (10.1)	91 (8.1)	98 (8.3)
Heart/circulatory problems	4 (5.8)	57 (5.1)	61 (5.1)
MS	0	50 (4.5)	50 (4.2)
Connective tissue disease	0	60 (5.4)	60 (5.1)
Gynaecological/hormonal	0	34 (3.0)	34 (2.9)
Bone/joint	2 (2.9)	30 (2.7)	32 (2.7)
Hypochondria	0	33 (3.0)	33 (2.8)
Cancer	2 (2.9)	27 (2.4)	29 (2.4)
Thyroid	2 (2.9)	27 (2.4)	29 (2.4)
Rheumatic fever	0	17 (1.5)	17 (1.4)
Lung related	4 (5.8)	17 (1.5)	21 (1.8)
Renal problems	2 (2.9)	18 (1.6)	20 (1.7)
Allergy	1 (1.4)	33 (3.0)	34 (2.9)
Gastro problems	1 (1.4)	13 (1.2)	14 (1.2)
Migraine	0	14 (1.3)	14 (1.2)
HSP	0	7 (0.6)	7 (0.6)
Polymyalgia	0	2 (0.2)	2 (0.2)
Age	0	9 (0.8)	9 (0.8)
Non-specified autoimmune	1 (1.4)	12 (1.1)	13 (1.1)
Epilepsy	0	9 (0.8)	9 (0.8)
APS	0	8 (0.7)	8 (0.7)
Gout	2 (2.9)	4 (0.4)	6 (0.5)
Hepatitis	0	3 (0.3)	3 (0.3)
Inflammatory condition	2 (2.9)	19 (1.7)	21 (1.8)
Other conditions	7 (10.1)	90 (8.1)	97 (8.2)
Patient stated “too many to mention”	1 (1.4)	13 (1.2)	14 (1.2)
No conclusion reached	1 (1.4)	14 (1.3)	15 (1.3)
Did not indicate	1 (1.4)	28 (2.5)	29 (2.4)

Supplementary Table 7: Main group of co morbidities by gender

Co-morbidity variable n (%)	Men<br>n=156	Women<br>n=2371	Total<br>n=2527
Thyroid problems	6 (3.8)	320 (13.5)	326 (12.9)
RA	14 (9.0)	269 (11.3)	283 (11.2)
Osteoporosis	10 (6.4)	217 (9.2)	227 (9.0)
Osteoarthritis	11 (7.1)	245 (10.3)	256 (10.1)
Heart	37 (23.7)	254 (10.7)	291 (11.5)
Diabetes	11 (7.1)	121 (5.1)	132 (5.2)
Cancer	14 (9.0)	92 (3.9)	106 (4.2)
Depression	5 (3.2)	83 (3.5)	88 (3.5)
Kidney	8 (5.1)	73 (3.1)	81 (3.2)
Cataracts	0	18 (0.8)	18 (0.7)
MS	1 (0.6)	10 (0.4)	11 (0.4)

*Percentages calculated from whole group. Only selected co-morbidities reported

Supplementary Table 8: Reported symptom frequently suffering from by gender

Symptom n (%)		Men n=156	Women n=2371	Total n=2527
Fatigue and weakness		133 (85.3)	2166 (91.4)	2299 (91.0)
Joint pain and/or swelling		92 (59.0)	1865 (78.7)	1957 (77.4)
Poor circulation or Raynauds		70 (44.9)	1473 (62.1)	1543 (61.1)
Back pain		59 (37.8)	1226 (51.7)	1285 (50.9)
Flu-like symptoms/night sweats		44 (28.26)	1238 (52.2)	1282 (50.7)
Headache/migraine		42 (26.9)	1160 (48.9)	1202 (47.6)
Ulcers		43 (27.6)	1098 (46.3)	1141 (45.2)
Rash		57 (36.5)	1054 (44.5)	1111 (44.0)
Breathlessness		73 (46.8)	947 (39.9)	1020 (40.4)
Depression		52 (33.3)	930 (39.2)	982 (38.9)
Hair-loss		28 (18.0)	850 (35.9)	878 (34.7)
Haematological problems		31 (19.9)	664 (28.0)	695 (27.5)
Kidney problems		35 (22.4)	406 (17.1)	441 (17.5)
Miscarriage		-	423 (17.8)	-
Stroke or mini stroke		12 (7.7)	172 (7.3)	184 (7.3)
Angina		12 (7.7)	127 (5.4)	139 (5.5)
Symptoms frequently suffering from	0	9 (5.8)	34 (1.4)	43 (1.7)
	1-5	89 (57.1)	797 (33.6)	886 (35.1)
	6-10	50 (32.1)	1310 (55.3)	1360 (53.8)
	11-16	8 (5.1)	230 (9.7)	238 (9.4)

Supplementary Table 9: Symptom most difficult to live with ranked in the top 3 by individual by gender

Symptom ranked in top 3	Men n=156	Women n=2371	Total n=2527
Fatigue and weakness	105 (67.3)	1939 (81.8)	2044 (80.9)
Joint pain and/or swelling	75 (48.1)	1452 (61.2)	1527 (60.4)
Poor circulation or Raynauds	28 (18.0)	413 (17.4)	441 (17.5)
Back pain	22 (14.1)	425 (17.9)	447 (17.7)
Flu-like symptoms/night sweats	10 (6.4)	289 (12.2)	299 (11.8)
Headache/migraine	15 (9.6)	430 (18.1)	445 (17.6)
Ulcers	9 (5.8)	173 (7.3)	182 (7.2)
Rash	23 (14.7)	227 (9.6)	250 (9.9)
Breathlessness	35 (22.4)	295 (12.4)	330 (13.1)
Depression	28 (18.0)	389 (16.4)	417 (16.5)
Hair-loss	7 (4.5)	186 (7.8)	193 (7.6)
Haematological problems	8 (5.1)	64 (2.7)	72 (2.9)
Kidney problems	12 (7.7)	90 (3.8)	102 (4.0)
Stroke or mini stroke	0	35 (1.5)	35 (1.4)
Angina	2 (1.3)	18 (0.8)	20 (0.8)
Miscarriage	-	13 (0.6)	-

NB 13 other symptoms ranked; 89 did not indicate

Supplementary Table 10: Condition that makes Lupus worse by gender

Condition	Men n=156	Women n=2371	Total n=2527
Over exertion/fatigue	108 (96.2)	1959 (82.6)	2067 (81.8)
Stress/worry	82 (52.6)	1784 (75.2)	1866 (73.8)
Sunlight	67 (43.0)	1365 (57.6)	1432 (56.7)
Cold/damp	70 (44.9)	1333 (56.2)	1403 (55.5)
Fluorescent lighting	31 (19.9)	596 (25.1)	627 (24.8)
Heat	28 (18.0)	734 (31.0)	762 (30.2)
Hormonal changes	-	507 (21.4)	-
Noise	21 (13.5)	353 (14.9)	374 (14.8)
Other	10 (6.4)	213 (9.0)	223 (8.8)

Supplementary Table 11: Present healthcare professional by gender group

Healthcare professional		Men n=156	Women n=2371	Total n=2527
Rheumatologist		104 (66.7)	1981 (83.6)	2085 (82.5)
Dermatologist		29 (18.6)	397 (16.7)	426 (16.9)
Nephrologists		30 (19.2)	255 (10.8)	285 (11.3)
Neurologist		6 (3.9)	190 (5.0)	196 (7.8)
Immunologist		7 (4.5)	96 (4.1)	103 (4.1)
Paediatrician		2 (13)	14 (0.6)	16 (0.6)
Number currently seeing	0	24 (15.4)	223 (9.4)	247 (9.8)
	1	91 (58.3)	1521 (64.2)	1612 (63.8)
	2	37 (23.7)	494 (20.8)	531 (21.0)
	3	3 (1.9)	110 (4.6)	113 (4.5)
	4	1 (0.6)	22 (0.9)	23 (0.9)
	5	0	0	0
	6	0	1 (0.04)	1 (0.04)
